# Medical profile of patients referred to an Australian postgraduate oral surgery clinic

**DOI:** 10.1002/cre2.781

**Published:** 2023-09-07

**Authors:** Anthony Chu Seng Ngu, Sitanshu Arora, Peter Reher

**Affiliations:** ^1^ School of Medicine and Dentistry Griffith University Southport Australia

**Keywords:** clinical pharmacology, dental education, multimorbidity, oral surgery

## Abstract

**Background and Objectives:**

Thorough knowledge of a patient's medical history and medications is necessary for providing safe oral surgery care, and may be considered a form of risk management. This study investigated the prevalence of medical conditions and medication types in patients referred to an Australian postgraduate oral surgery clinic over 2 years.

**Materials and Methods:**

A retrospective cross‐sectional study of the clinical records of 233 randomized patients referred to the Griffith University (Queensland, Australia) postgraduate oral surgery clinic in 2018 and 2019 was performed. Medical conditions and medications were counted and categorized, and descriptive statistics were generated.

**Results:**

In all, 133 patients (57%) had at least one medical condition. 58% of them (77) had two or more categories of medical conditions, representing nearly a third (33.0%) of all sampled patients. The most prevalent category of medical conditions was psychiatric (25.3%), followed closely by cardiovascular (24.5%) diseases. Cardiovascular medications were the most prevalent, comprising 23.6% of all medications recorded, followed by psychotropics (18.3%).

**Conclusion:**

Over half of patients referred to the postgraduate oral surgery clinic had at least one systemic medical condition. Nearly a third of patients referred had at least two distinct systemic medical conditions. With an ageing population and the accompanying rise in multimorbidity globally, dental school curricula must adapt to prepare students to meet these challenges in their careers.

## 1 INTRODUCTION

Thorough knowledge of a patient's medical history and medications is necessary to provide safe oral surgery care (Moore et al., 2019). Predictable hemostasis, healing, and pain control are desirable aspects of any surgical procedure. Awareness of the implications medical conditions and medications have on patient wellbeing enables the appropriate management of complications that may arise during and after treatment or their prevention entirely (DeAngelis et al., 2010). These include medical emergencies brought about by the inherent stresses of oral surgery procedures (Zachar & Reher, 2022). Hence, detailed knowledge of a patient's medical history can be considered a form of risk management in itself (Patel et al., 2014).

Students undertaking their primary dental degree have been observed to be lacking adequate knowledge over the medical conditions of their patients and managing them appropriately in the oral surgery setting (Dennis et al., 2017; Emam et al., 2018). It is therefore not surprising that complex patient medical histories are commonly cited reasons for referral to more experienced clinicians (Arora et al., 2023). This naturally extends into their careers as general dental practitioners, especially those without further training in minor oral surgery (Cottrell et al., 2007; Coulthard et al., 2000). The rise of multimorbidity is likely exacerbating trepidation toward providing minor oral surgery care to medically compromised patients in the primary care setting (Watt & Serban, 2020).

Studies examining the medical profiles of patients undergoing minor oral surgery procedures at university teaching settings have been carried out in South Asia (Kolte et al., 2014; Mahmood et al., 2020; Natarajan et al., 2019; Santhosh, 2020; Santhosh Kumar, 2021; Santhosh Kumar & Rajan, 2016; Siddiqi et al., 2016; Walia et al., 2017) and Western Europe (Amado Cuesta et al., 2004). To date, this is the first study in the southern hemisphere specifically investigating the prevalence of medical conditions and medication types in patients referred to postgraduate oral surgery students.

## 2 MATERIALS AND METHODS

Data collection was carried out retrospectively by a single reviewer (ACS Ngu). A macro was used in Microsoft® Excel® (Microsoft Corp.) to randomly select 233 of the 587 patients seen at the Griffith University postgraduate oral surgery clinic in the 2‐year period of 2018 and 2019. Sample size calculators (Momentive Inc., 1999; Qualtrics, 2021) deemed this figure to provide a representative sample with a confidence interval of 95%.

Data were collected from a combination of patient registration forms, medical history pro formas, referral letters, and clinical notes saved on the Titanium® (Titanium Solutions Ltd.) patient management software. Data included did not extend beyond 18 months before the date of first presentation at the postgraduate oral surgery clinic, accounting for the fact that the clinic does not run every week of the year due to the academic calendar.

Medical conditions were recorded under categories as defined by Patel et al. (2014) Classifying diseases under these categories was done according to the *International Classification of Diseases for Mortality and Morbidity Statistics 11th Revision* (ICD‐11) (World Health Organization, 2019). The presence or absence of drug allergies and habits, such as alcohol consumption, cigarette smoking, and recreational drug use, was also recorded. Medications were listed and categorized according to the latest *Australian Medicines Handbook* (Australian Medicines Handbook, 2021). Demographic data collected included patient gender and age at the time of being first seen at the clinic. Where appropriate, variables were numerically coded and entered in a Microsoft® Excel® spreadsheet and descriptive statistics generated using the same software.

Patients who were not actually seen at the postgraduate oral surgery clinic, but somehow included in the pool of 587 patients, were excluded from the study. Ethics approval was obtained under the registration number 2019/119 through the Griffith University Human Research Ethics Committee.

## 3 RESULTS

Of the 233 sample patients, 51.9% (121) were females and 48.1% (112) males. The mean age was 40.1 years, with a range of 9–89 years. Nearly a third (32.6%) of the sample were aged between 25 and 44, with the next largest portion of patients aged between 15 and 24.

**Figure 1:**
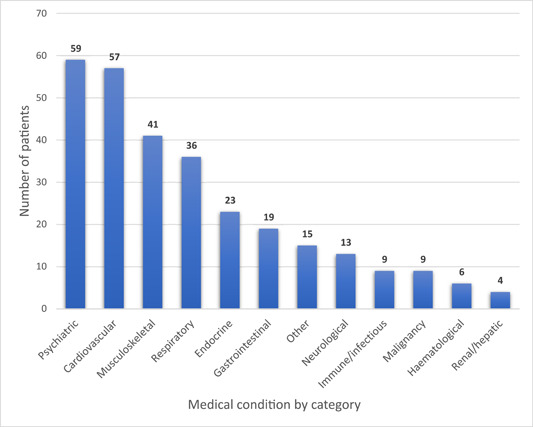
The 10 most prevalent categories of medical conditions in the sample population in descending order (*n* = 233).

57% (133) of patients had at least one medical condition, and 58% of them (77) had two or more categories of medical conditions, representing approximately a third (33.0%) of all sample patients (Table 1). Psychiatric conditions comprised the most prevalent medical condition category (25.3%), followed closely by cardiovascular (24.5%) diseases (Figure 1).

**Table 1:** Demographic characteristics of patients in sample population at the time of first presentation to the Griffith University postgraduate oral surgery clinic (*n* = 233).

Characteristic	Number of patients (%)
Age (years)	
≤14	3 (1.3)
15–24	68 (29.2)
25–44	76 (32.6)
45–64	45 (19.3)
≥65	41 (17.6)
Sex	
Female	121 (51.9)
Male	112 (48.1)
Number of categories of medical conditions	
Nil	100 (42.9)
One	56 (24.0)
Two or more	77 (33.0)
Drug allergies	
Nil	181 (77.7)
Positive	50 (21.5)
Not recorded	2 (0.9)
Alcohol consumption	
Yes	146 (62.7)
No	76 (32.6)
Not recorded	11 (4.7)
Cigarette smoking	
Positive history	105 (45.1)
Never smoked	124 (53.2)
Not recorded	4 (1.7)
Recreational drug use	
Positive history	13 (5.6)
Never used	97 (41.6)
Not recorded	123 (52.8)

Alcohol consumption was the most frequently reported substance use behavior, followed by cigarette smoking. Recreational drug use history was recorded in less than half of the sample patients (Table 1).

Cardiovascular medications were the most prevalent, comprising 23.6% of all medications recorded, followed by psychotropics, which encompassed 18.8% of all medications (Table 2). When grouped more specifically according to therapeutic indication, antihypertensives were the most prevalent medication class, followed by antidepressants (Table 3). Twenty‐eight patients (12.1%) were on antithrombotic medications (Tables 2 and 3). Seven, representing exactly a quarter of them, were on direct oral anticoagulants (DOACs) while three (10.7%) were warfaranised. A history of antiresorptive therapy was present in 14 patients (6.0%). Six (2.6%) patients were on immunosuppressant therapy.

**Table 2:** Top 10 drug classes by anatomical/physiological system (*n* = 440).

Drug class by anatomical/physiological system	Count	Prevalence in sample (%)
Cardiovascular	104	23.6
Psychotropic^a^	83	18.8
Analgesics	44	10.0
Endocrine	35	8.0
Blood and electrolyte	29	6.6
Complementary medicines^b^	32	7.3
Respiratory	25	5.7
Gastrointestinal	22	5
Obstetric and gynaecological	20	4.5
Immunomodulators and antineoplastics	16	3.6

^a^ Including certain antiepileptics that mood‐stabilising effects (i.e., carbamazepine, lamotrigine, and valproate).

^b^ Including vitamin and mineral supplements.

**Table 3:** Top 10 drug classes by therapeutic indication (*n* = 440).

Drug class by therapeutic indication	Count	Prevalence in sample (%)
Antihypertensives	74	16.8
Antidepressants	53	12.0
Complementary medicines^a^	32	7.3
Drugs for asthma and chronic obstructive pulmonary disease	25	5.7
Non‐opioid analgesics	24	5.5
Drugs for dyslipidemia	20	4.5
Opioid analgesics	20	4.5
Drugs for contraception	19	4.3
Antiplatelets	18	4.1
Antiepileptics^b^	16	3.6

^a^ Including vitamin and mineral supplements.

^b^ Including carbamazepine, lamotrigine, and valproate, but excluding benzodiazepines.

The presence or absence of drug allergies was recorded in 231 (99.1%) patients, of whom 50 (21.5%) reported an adverse drug reaction (Table 1). Half of the patients who reported a drug allergy did so regarding analgesics, most of which were to opioids (*n* = 19, 78.0%) and a quarter nonsteroidal anti‐inflammatory drug (NSAIDs). Antibiotics were the second most common medication class that had adverse reactions reported (*n* = 22, 44.0%), with penicillin comprising over three‐quarters (*n* = 17, 77.3%) of them in this group. Seven patients (14%) reported being allergic to both an analgesic and antibiotic. Altogether, patients who reported allergies to antibiotics and analgesics represented 80% of all patients who reported any drug allergy.

## 4 DISCUSSION

### 4.1 Prevalence

The overall prevalence of medical conditions in this study was 57.1% (Table 1 and Figure 1), which was notably higher than that in the Australian population (47%) of the comparative time period (2017–2018) (Australian Bureau of Statistics, 2017–2018). The prominence of psychiatric conditions in this study's population paralleled that of Queensland (the state where the university's clinic is situated) then and nationally. Queensland also had the highest prevalence of psychiatric conditions (22.7%) in Australia, with an overall prevalence of 20.1% (Australian Bureau of Statistics, 2022, 2018). Tellingly, data (Kisely et al., 2015, 2021; Kisely & Lalloo, 2021) from the state's hospitals revealed that dental conditions accounted for the third most common reason for hospitalization in people with psychiatric conditions, and people with a history of psychiatric treatment were 72% more likely than the rest of the population to present to emergency departments with an avoidable dental condition.

The prevalence of psychiatric conditions in this study differs markedly from the results of the other studies that specifically examined patient medical profiles from outpatient oral surgery services in university settings, mainly in South Asia (Amado Cuesta et al., 2004; Kolte et al., 2014; Mahmood et al., 2020; Natarajan et al., 2019; Santhosh, 2020; Santhosh Kumar, 2021; Santhosh Kumar & Rajan, 2016; Siddiqi et al., 2016; Walia et al., 2017). Cardiovascular disease was generally the most prevalent disease category in them, and psychiatric conditions were barely mentioned, if at all. In fact, only two patients with psychiatric conditions were recorded in one study from India (Kolte et al., 2014). The dearth of psychiatric conditions in these studies may reflect structural and cultural attitudes toward their reporting as mental health conditions, including addiction disorders, comprise a significant, if not, the largest proportion of global disease burden (Rehm & Shield, 2019; Wainberg et al., 2017; Vigo et al., 2022). As a well‐established correlation exists between poor oral health and psychiatric illness, patients with psychiatric conditions can be expected to require oral surgery services on a higher basis than the general population (Macnamara et al., 2021).

### 4.2 Multimorbidity

Multimorbidity is formally defined by the World Health Organization (World Health Organization, 2016) as “the coexistence of two or more chronic health conditions in the same individual.” It is preferable as a term and concept to comorbidity, which describes the coexistence of other health conditions with an index condition that is the focus of attention. It reflects the shift toward patient‐centred care, whereby the patient's experience, priorities, and overall treatment burden are prioritized accordingly (Harrison et al., 2021). Approximately a third (33.0%) of all patients in this study could be considered to be multimorbid, constituting over half (57.9%) of all patients with medical conditions. This was a higher prevalence than that in the general Australian population (20%) in 2017–2018 (36) and likely reflects the aggregation of referrals for medically complex patients to the postgraduate oral surgery clinic, as well as the intersection of the socioeconomic determinants of health, multimorbidity, and poor oral health (Arora et al., 2023; Frydrych et al., 2020; Watt & Serban, 2020).

An increase in multimorbidity is accompanying the general trend of ageing populations in the developed world that has resulted from advances in healthcare and public health (Gill et al., 2020). Understandably, the treatment burden endured by patients with multimorbidity means oral health maintenance is often a low priority (Serban et al., 2019). It is therefore not unreasonable to expect the need for oral surgery services for increasingly frail and medically complex patients to grow, along with the challenges of providing safe and timely care. Students must be prepared as clinicians to navigate complex medical care plans involving multiple therapies drawn up by often disparate healthcare providers, and meet patient expectations that their treating practitioners will be able to manage medical emergencies in the dental setting competently (Vaughan et al., 2019; Watt & Serban, 2020).

Moves to improve health record accessibility for health care providers have been made by integrated electronic health records. To date, uptake of the Australian effort (My Health Record) has been haphazard for a variety of reasons. These include public concerns over privacy, data security, and simply a lack of awareness of its existence (Holt et al., 2023).

### 4.3 Pharmacology, pharmacovigilance and pharmacotherapeutics

Pharmacological knowledge is essential in appreciating the implications a patient's medical conditions may have on treatment planning in oral surgery, and dentistry in general. Insight into a patient's medication history facilitates safe dental interventions through risk stratification. A patient with a history of solely oral bisphosphonates for osteoporosis, for example, is at considerably lower risk of developing medication‐related osteonecrosis of the jaw (MRONJ) than one on monthly antiresorptive therapy for cancer with bony metastases (Ruggiero et al., 2022). Awareness of the varied indications for antithrombotic therapy and contemporary trends in anticoagulation therapy enables bleeding susceptibility to be discussed within the context of oral surgical practice (Kaplovitch & Dounaevskaia, 2019). The latter was evident in our study's findings, as more than twice the number of warfarinised patients were on DOACs and for conditions ranging from factor V Leiden thrombophilia and symptomatic relief for Raynaud's phenomenon.

There is a growing recognition for the need to incorporate pharmacotherapeutics and pharmacovigilance in the curriculum of healthcare professionals, including dentists, at undergraduate and postgraduate levels (Herrera Comoglio, 2020; Reumerman et al., 2018). Pharmacovigilance involves the detection, assessment, understanding, and prevention of adverse effects or any other drug‐related problem (World Health Organization, 2002). It is particularly topical for contemporary oral surgery, and indeed general dental practice, amidst the coinciding increase in multimorbidity and polypharmacy (Sinnott & Bradley, 2015; Watt & Serban, 2020). Over a fifth of patients (Table 1) in our study reported an adverse drug reaction, with analgesics and antibiotics the top two featured drug groups. Of concern was the observation that only five of the 17 patients who reported penicillin allergies had their past reactions recorded, and three were of urticaria. This poses obvious challenges for dental students upon graduating to prescribe safely and with confidence, which has been noted in the Australian literature (Park et al., 2023; Teoh et al., 2019).

The application of pharmacology, pharmacotherapeutics, and pharmacovigilance in clinical dental practice is aptly illustrated through the psychiatric patient experience. Xerostomia, for instance, is a commonly encountered oral side effect of their medications and may accelerate caries progression and the rate of tooth loss (Cockburn et al., 2017). Antidepressants and antipsychotics are associated with a higher risk of developing alveolar osteitis after dental extractions (Parthasarathi et al., 2011). Achieving safe and effective analgesia is an important consideration, as these medications may induce gastrointestinal side effects that can be exacerbated by nonselective NSAIDs (Oliva et al., 2021). Patients on selective serotonin reuptake inhibitors (SSRIs) or serotonin and norepinephrine reuptake inhibitors (SNRIs) may find codeine and tramadol to be of limited analgesic utility, as their conversion into active metabolites is impeded by the inhibition of cytochrome 2D6 (CYP2D6) enzymes by these antidepressants (Cazet et al., 2018). Simultaneous use of tramadol with an SSRI or SNRI also increases the risk of serotonin syndrome (Perananthan & Buckley, 2021). The analgesic potential of selective COX‐2 inhibitor NSAIDs (e.g., celecoxib) in patients on psychotropics appears to be understated.

### 4.4 Study limitations

Data collection and analysis was carried out by a single person who collated data from medical histories taken by different people with varying levels of clinical and academic experience. Consequently, an unknown degree of misclassification error regarding medical condition and medication categorization may have occurred. Recall bias by patients was also likely. However, data collected was checked and verified multiple times, and the accuracy and validity of the results are supported by their comparability to official state and national data. The decision to restrict this study's timeframe to 2 years of normal clinical functioning, intended to prevent distortion of data from COVID‐19‐related disruptions in 2020, means that the pre‐COVID‐19 era findings may not be directly translatable to future patient populations presenting for minor oral surgery care.

Nonetheless, it can be anticipated that the leading categories of medical conditions revealed in this study will likely remain similar for the foreseeable future. Unintended consequences of public health pandemic measures included delayed presentation and management of acute medical issues, and the postponement of elective surgery (Holt et al., 2020). Elevated rates of anxiety and depression symptoms have been attributed to pandemic‐induced impairments in work and social functioning globally (Bower et al., 2023). In Australia, a temporary halt to immigration and likely short‐term fall in fertility is expected to lead to a moderate increase in the proportion of older age groups in the overall population (Wilson et al., 2022). Altogether, these factors may contribute to an increase in multimorbidity and severity of chronic medical conditions in the long term, raising challenges to the safe delivery of oral surgery and dental care in general. The findings of this study thus remain relevant to dental education in the wake of the COVID‐19 pandemic.

## 5 CONCLUSION

This appears to be the first study specifically examining the medical profiles of patients referred to a university postgraduate oral surgery clinic in the southern hemisphere. Over half of patients referred to the Griffith University postgraduate oral surgery clinic in 2018 and 2019 had at least one medical condition and approximately a third were experiencing multimorbidity. Psychiatric, cardiovascular, musculoskeletal, respiratory, and endocrine conditions were the five most prevalent categories of medical conditions present. Cardiovascular medications were the most ubiquitous class of medications, followed by psychotropics. To help prepare dental students for safe and effective clinical practice, dental school curricula must increase their contemporary medical and pharmacological content.

## AUTHOR CONTRIBUTIONS

Anthony Chu Seng Ngu and Peter Reher conceived and designed the project. Anthony Chu Seng Ngu acquired the data. Anthony Chu Seng Ngu analyzed and interpreted the data. Sitanshu Arora, Anthony Chu Seng Ngu, and Peter Reher wrote the paper.

## CONFLICT OF INTEREST STATEMENT

The authors declare no conflict of interest.

## ETHICS STATEMENT

This study was able to proceed under GU Ref: No: 2019/119 approved by the Griffith University Human Research Ethics Committee.

## Data Availability

Data sharing is not applicable—no new data were generated.
